# Supplementary material for: Differential microRNA and Target Gene Expression in Scots Pine (Pinus sylvestris L.) Needles in Response to Methyl Jasmonate Treatment
Source: Genes (Basel). 2024 Dec 27;16(1):26. doi: 10.3390/genes16010026 (PMC11765084; doi:10.3390/genes16010026)
Supplement: Supplementary file 1 [file genes-16-00026-s001.zip › Supplementary_file5.pdf]

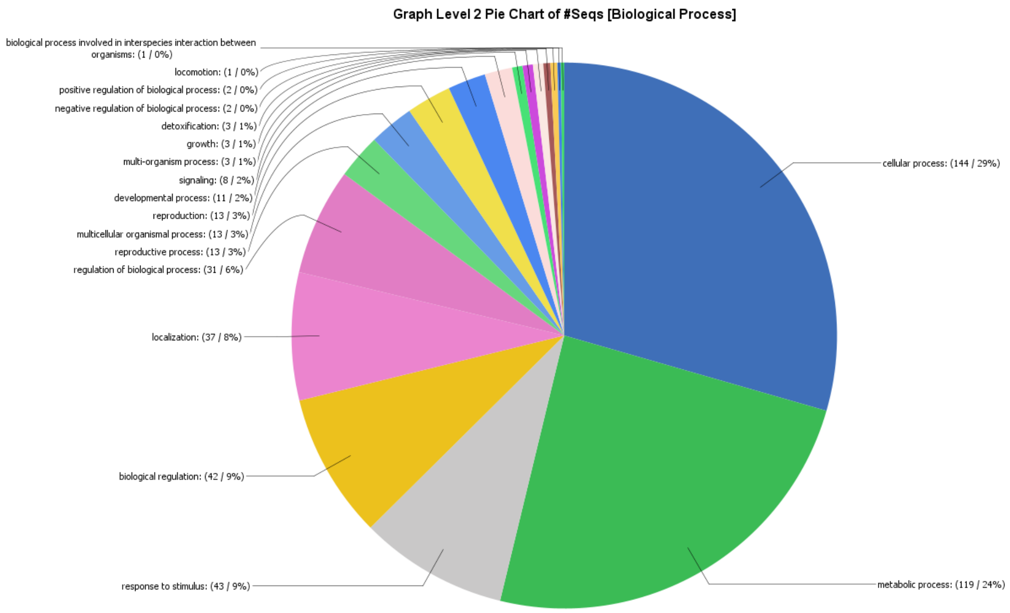


**Figure S1**. Distribution of DE up-regulated miRNA target genes according to GO category (biological process)

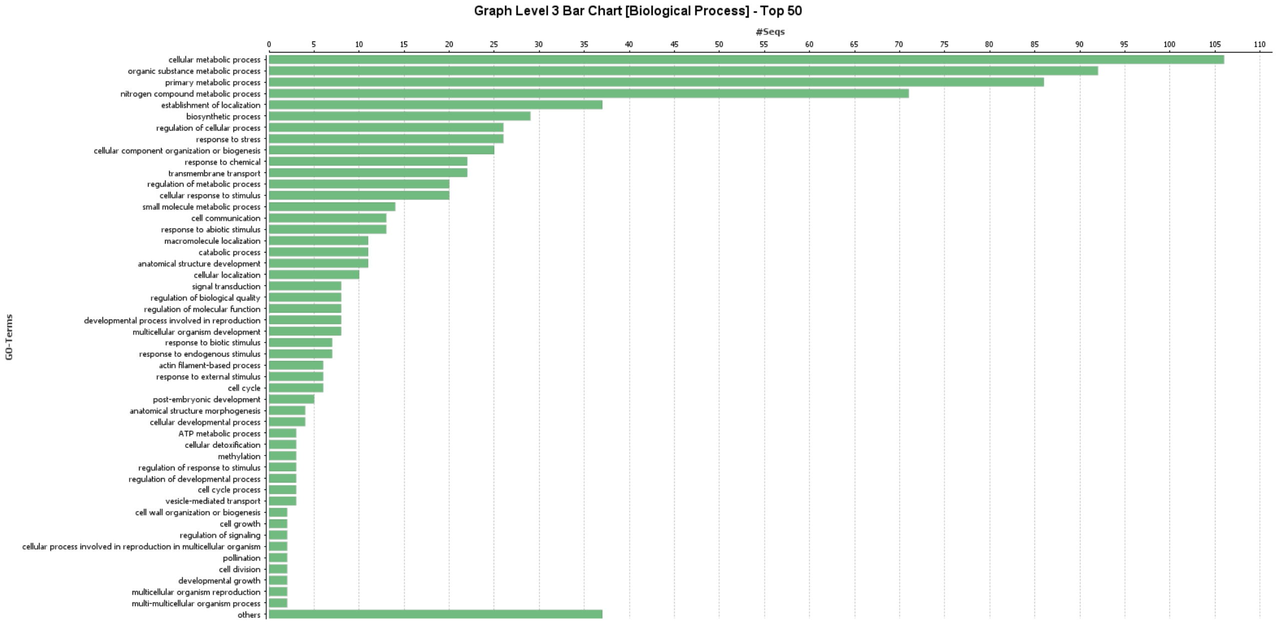


**Figure S2**. Distribution of DE up-regulated miRNA target genes within GO category (biological process) response to stimulus using non-redundant protein database

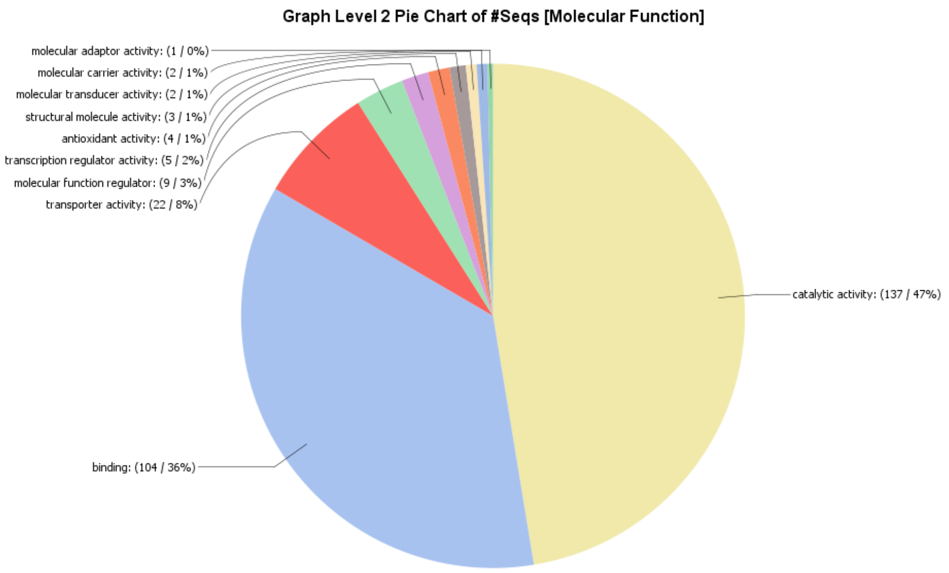


**Figure S3**. Distribution of DE up-regulated miRNA target genes according to GO category (molecular function)

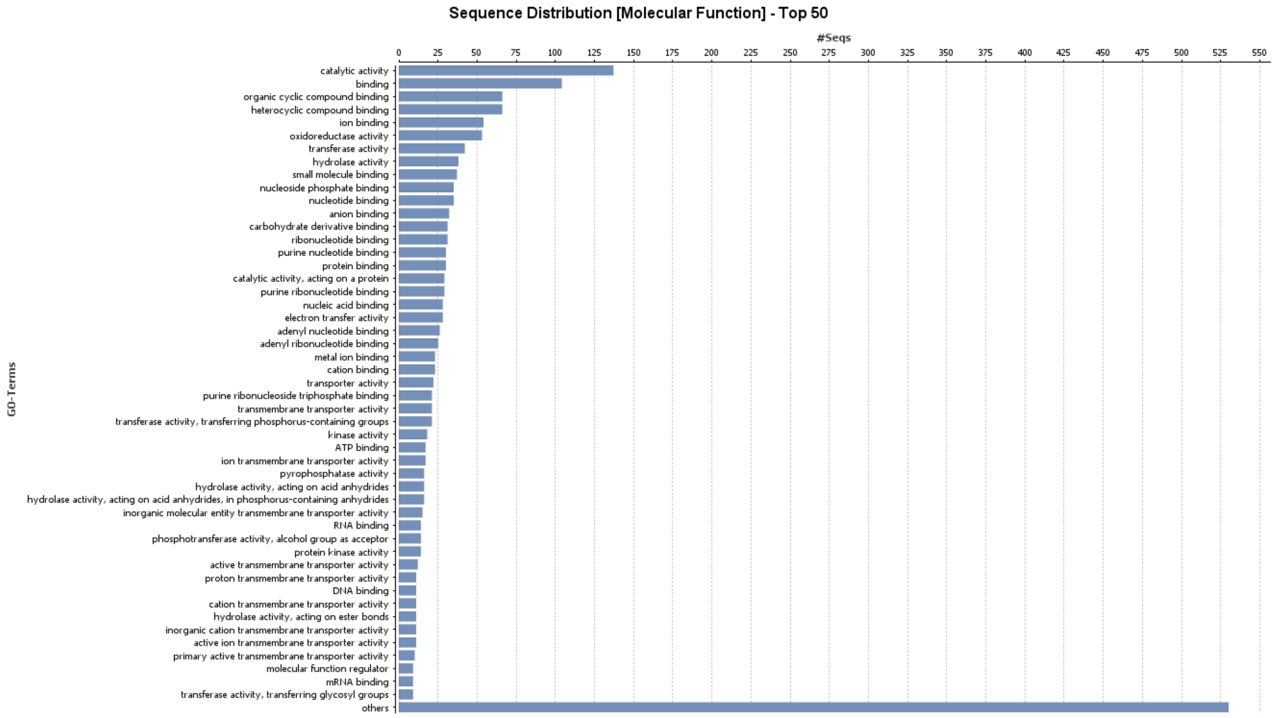


**Figure S4.** Distribution of DE up-regulated miRNA target genes according to GO category (molecular function) using non-redundant protein database

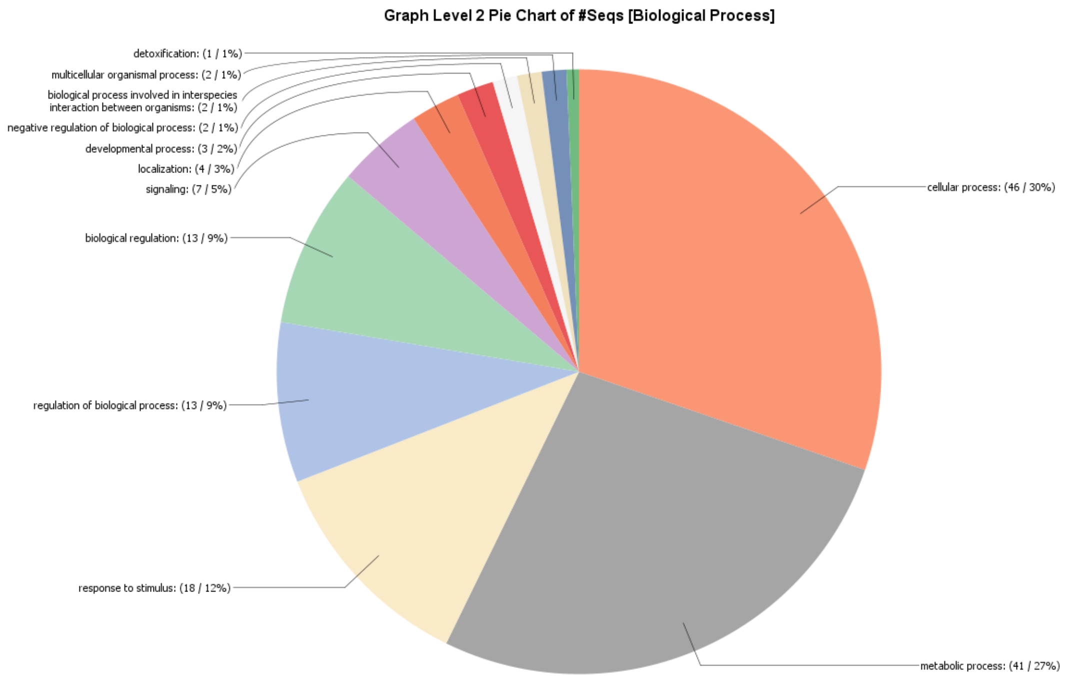


**Figure S5**. Distribution of DE down-regulated miRNA target genes according to GO category (biological process)

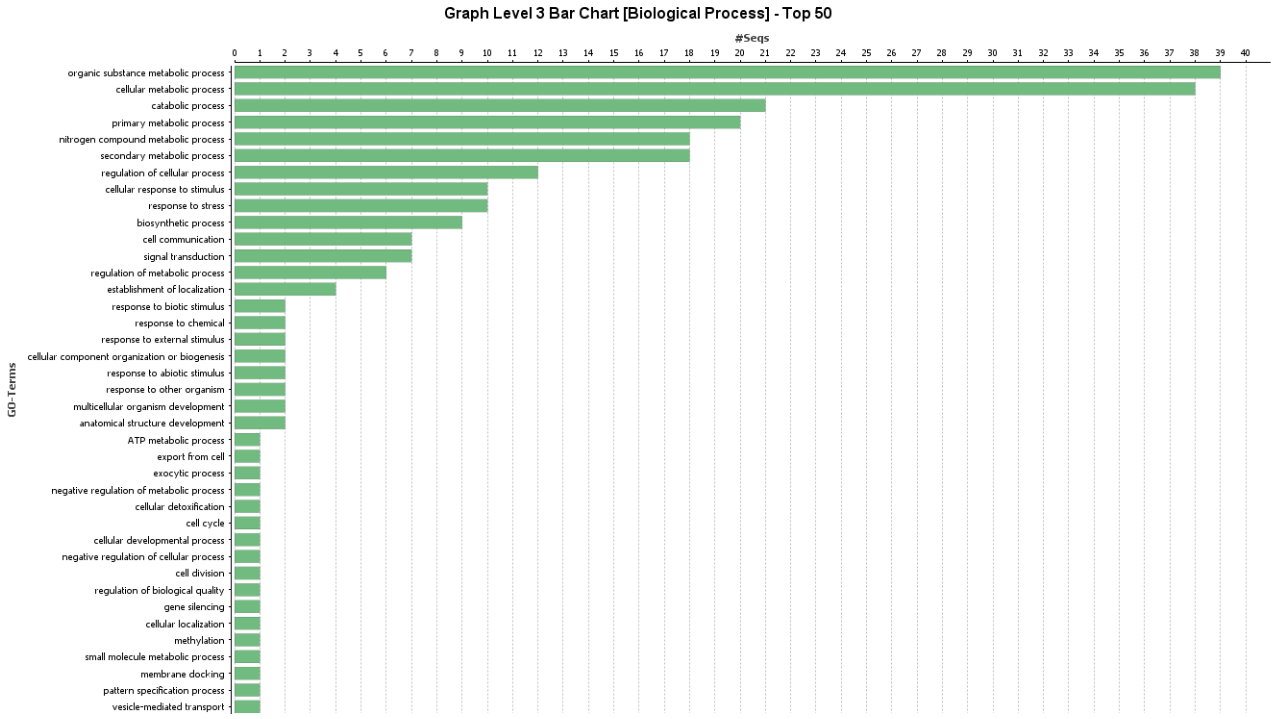


**Figure S6.** Distribution of DE down-regulated miRNA target genes within GO category (biological process) response to stimulus using non-redundant protein database

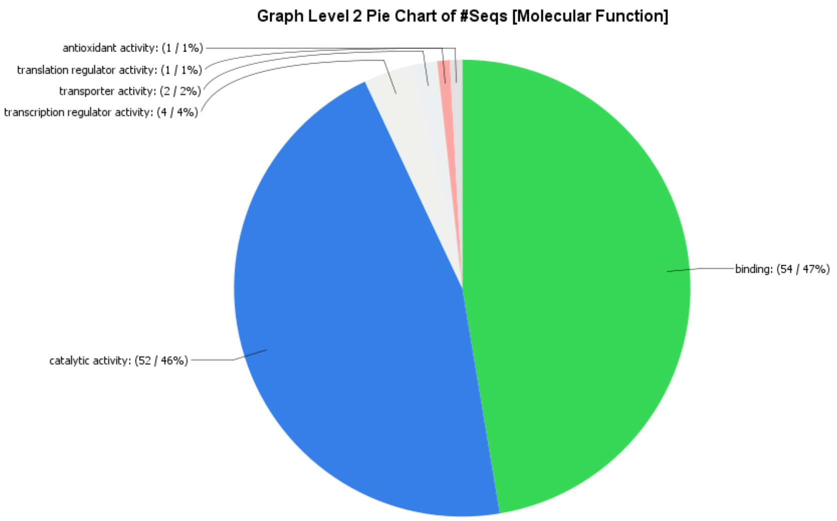


**Figure S7.** Distribution of DE down-regulated miRNA target genes according to GO category (molecular function)

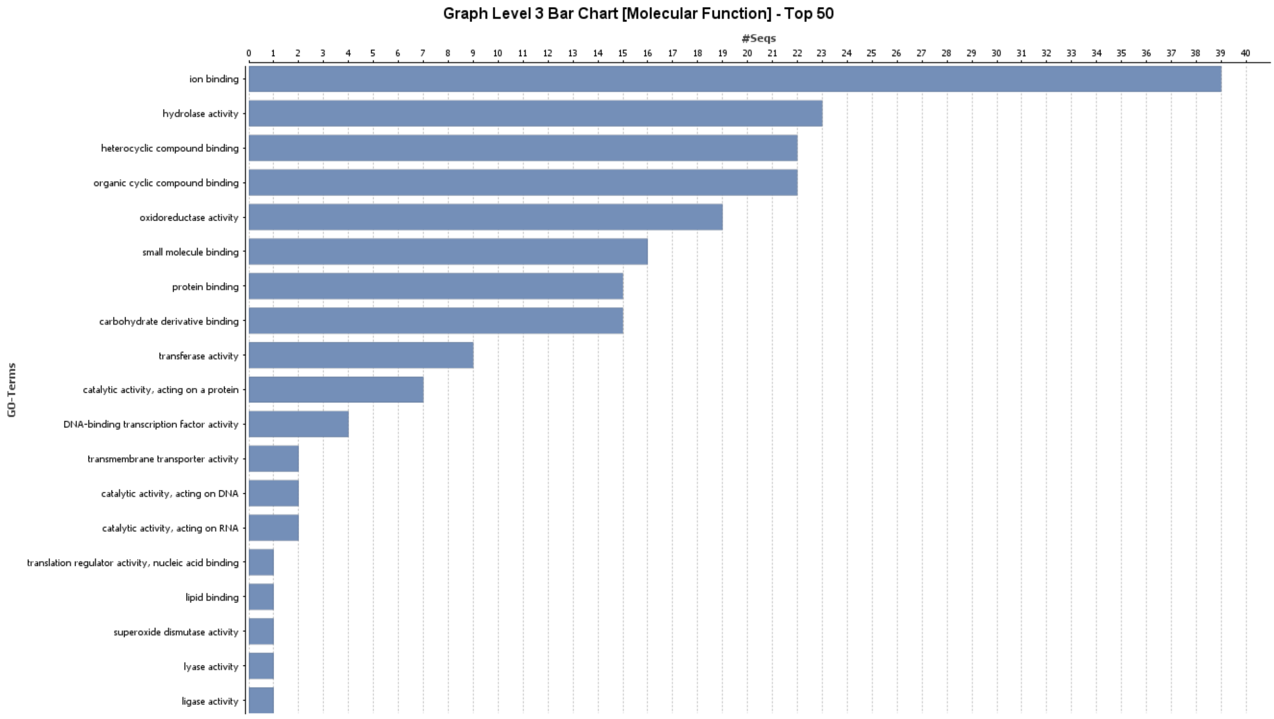


**Figure S8**. Distribution of DE down-regulated miRNA target genes according to GO category (molecular function) using non-redundant protein database
